# Predicting long-term response to strong opioids in patients with low back pain: findings from a randomized, controlled trial of transdermal fentanyl and morphine

**DOI:** 10.1186/1741-7015-5-39

**Published:** 2007-12-21

**Authors:** Eija Kalso, Karen H Simpson, Robert Slappendel, Joachim Dejonckheere, Ute Richarz

**Affiliations:** 1 Pain Clinic, Helsinki University Central Hospital, Helsinki, Finland; 2 Leeds Teaching Hospitals, Leeds, UK; 3 Sint Maartenskliniek, Nijmegen, The Netherlands; 4 SGS, Mechelen, Belgium; 5 Janssen-Cilag, Baar, Switzerland

## Abstract

**Background:**

Some patients with long-standing low back pain will benefit from treatment with strong opioids. However, it would be helpful to predict which patients will have a good response. A fixed-term opioid trial has been recommended, but there is little evidence to suggest how long this trial should be. We assessed data from a large-scale randomized comparison of transdermal fentanyl (TDF) and sustained-release oral morphine (slow-release morphine; SRM) to determine characteristics of treatment responders.

**Methods:**

This was a secondary analysis of a previously published 13-month randomized trial involving 680 patients with long-standing low back pain (median age 52 years, 61% women, median duration of back pain 87 months). Pain relief was recorded using visual analogue scales (VAS). Treatment response was defined as pain relief of at least 30% from baseline to any point during the trial. We used a step-wise logistic regression to identify variables that might predict response to treatment. Covariates included treatment group, sex, age, duration of pain, presence of neuropathic pain, baseline pain scores, educational/employment status, use of high doses of opioids, and social functioning (SF)-36 scores.

**Results:**

Over half the patients in both groups (n = 370; 54% TDF, 55% SRM) were treatment responders. There were no differences between the TDF and SRM responders in terms of age, sex, type or duration of pain between responders and non-responders. The difference in response to treatment between responders and non-responders could be detected at 3 weeks. Lack of response after 1 month had a stronger negative predictive value (i.e., ability to detect non-responders) than the presence of response after 1 month. The most influential factors for predicting a response were employment status (χ^2 ^= 11.06, p = 0.0259) and use of high doses of opioids (χ^2 ^= 3.04, p = 0.0811).

**Conclusion:**

No clear pattern of baseline pain (type or severity) or patient characteristics emerged that could be used to predict responders before the start of opioid treatment. However, a 1-month trial period appears sufficient to determine response and tolerability in most cases.

## Background

Strong opioids are accepted as an option for patients with long-standing low back pain, but not all patients respond satisfactorily to this treatment. It would be helpful to be able to predict which patients are most likely to have a good, long-term response to strong opioid therapy to avoid exposing patients to ineffective treatments or side-effects. Even if it does not prove possible to identify good responders before starting treatment, it would be useful to know how long an opioid trial is required to determine response. We have analyzed findings from a randomized, controlled trial of transdermal fentanyl and sustained-release oral morphine in strong-opioid naive patients with chronic low back pain to gather information about predicting response and about the inter-relationships between analgesia and other effects of treatment.

## Methods

The primary study (FEN-INT-26) has been published separately [1]. It included 680 patients with long-standing low back pain who were randomized to treatment with either slow-release morphine (SRM) or transdermal fentanyl (TDF) for 13 months. Individuals who had received regular treatment with a strong opioid (i.e. more than four doses over a 7-day period) at any time during the 4 weeks preceding the study were excluded.

Patients were excluded if they had conditions that were likely to predispose them to ventilatory depression, intolerance to morphine or fentanyl (e.g. chest disease, renal dysfunction), skin problems that might affect transdermal delivery), a history of alcohol or substance abuse, or a life-limiting condition.

Pain relief was assessed weekly using visual analogue scales for pain relief (VAS) from patient diaries. There were monthly assessments of pain (at rest, on movement, during the day and at night). Adverse events were recorded throughout the study. For the purpose of this secondary analysis, treatment response was defined as pain relief of at least 30% from baseline to any point during the trial [2].

We performed a step-wise logistic regression to identify variables that might predict response to treatment with strong opioids during the trial. Covariates included in the initial model were: randomization group (fentanyl or morphine), sex, age, duration of pain, presence of neuropathic pain, educational/employment status, use of high doses of opioids (>100 μg/h TDF or >390 mg/day SRM) during the trial, baseline scores for pain at rest, pain on movement, and SF-36 scores (mental health, social functioning and physical functioning). The selection of covariates focused on those, which might have a possible effect on treatment outcome based on published literature and clinical experience [3-12]. We examined the effects of these variables on the likelihood of patients experiencing at least a 30% or 50% pain relief at several time points. Responses at earlier time points (1 month) were analyzed as predictors for response later on.

### Patient characteristics

The median age was 52 years (range 20–89), 61% of the 680 participants were women. The median duration of back pain was 87 months (range 2–768, mean 125 months); 51% of patients completed the study. Reasons for withdrawal included adverse events (37% TDF, 31% SRM) and insufficient response (5% TDF, 4% SRM).

## Results

### Responder analysis

Treatment response was defined as a pain relief of at least 30% (VAS scores) at any time point during the trial. Over half the patients in both groups (n = 370; 54% for TDF, 55% for SRM) were classified as treatment responders. There were no differences between the TDF and SRM responders in age, sex, type of pain (nociceptive or neuropathic) or duration of pain between responders and non-responders. The physical functioning score of the SF-36 differed only slightly between responders (28, 3) and non-responders (30, 1).

Of the participants who experienced at least a 30% pain relief at any time during the trial, most (74% for TDF and 70% for SRM) also experienced at least a 50% reduction in pain severity in one of the back pain categories. There was no difference between the 50% responders and non-responders in baseline characteristics.

The difference in response to treatment between responders and non-responders could be detected at 3 weeks. Both groups had similar pain severity after 1 week, but by 3 weeks the non-responders had, on average, more pain than at baseline, while the responders had less pain. This suggests that an assessment period of 1 month may be sufficient to determine responsiveness to a strong opioid.

**Figure 1:**
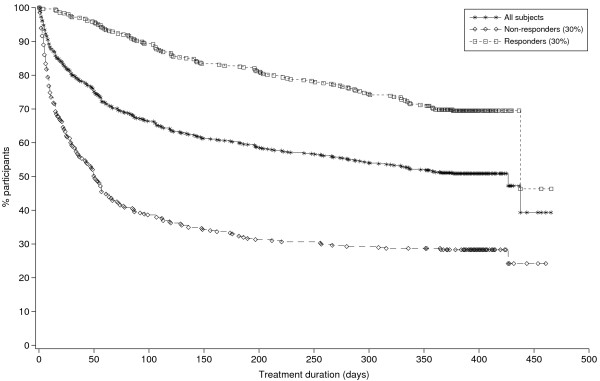
Treatment duration in responders and non-responders.

The amount of patients suffering from moderate to severe pain in any of the low back pain categories was lower in the responder group at endpoint compared to the non-responder group (Figure 1). There was however also a small reduction of patients suffering from moderate to severe pain in any of the low back pain categories in the non-responder group compared to baseline.

The odds ratios for the likelihood of being classified as a responder at 6 and 12 months (depending on whether patients were responders at 1 month) are shown in Table 1. These indicate that a lack of response after 1 month has a stronger negative predictive value (i.e. ability to detect non-responders) than the presence of response after 1 month. The predictive value for 50% pain relief was slightly higher than for 30% pain relief (odds ratios 5.08 and 4.36, respectively), but both were significantly associated with response at 6 months (p < 0.0001 in both cases). Response at 1 month was also associated with response at 12 months, although the odds ratios were less pronounced at this stage, probably due to the larger number of missing cases (OR 3.17 (95% CI 1.77–5.68) for 30% response and 3.47 (1.30–9.28) for 50% response).

**Table 1:** Relationship between pain relief at 1 month and at 6 months

**Response at 1 month**	**Response at 6 months n (%)**			**Odds ratio (95% CI)**
		
	**No**	**Yes**	**Missing**	
**30% Response**				

**No**	188 (55)	49 (14)	105	4.36 (2.61–7.28)
**Yes**	44 (35)	50 (40)	30	

**50% Response**				

**No**	261 (62)	36 (9)	105	5.08 (2.36–10.93)
**Yes**	14 (29)	20 (42)	14	

**Figure 2:**
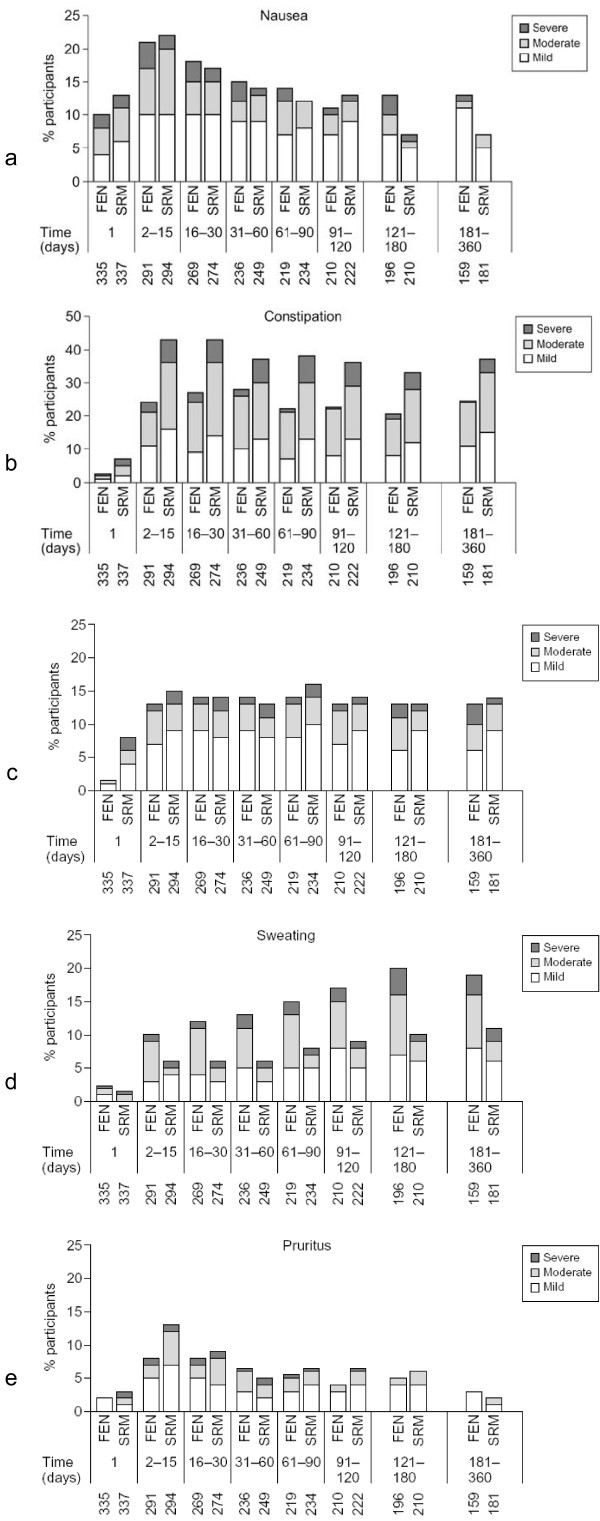
**Incidence and severity of (a) nausea, (b) constipation, (c) somnolence, (d) sweating, and (e) pruritus**. FEN, patients receiving transdermal fentanyl; SRM, patients receiving sustained release oral morphine. 1, incidence at day 1; 2–15, incidences at day 2, 3, etc. Numbers below x-axis show number of patients at each time point.

Treatment responders were more likely to complete the study than non-responders (69% for 30% responders vs 28% for non-responders). The most common reason for non-completion was an adverse event. On average, the non-responders dropped out of the study significantly earlier than the treatment responders (54 days vs 178 days p < 0.001, Figure 2). The rate of dropout for non-responders appeared to be greatest during the first 2 months, after which it reduced.

The dose pattern from 4 weeks to endpoint was broadly similar for 30% responders and non-responders who completed the study. The most common category of dose change over this period was 75–100%; this was observed in 32% of responders and 35% of non-responders who completed the study. The proportion of patients whose opioid dose more than doubled between 4 weeks and study end (for those who completed the study) was identical for responders and non-responders (24% in both groups).

### Characteristics of non-responders

About half the participants were classified as treatment responders (i.e. had at least a 30% pain relief VAS AUC [Area under the curve]); over three-quarters of participants (530/680 = 78%) experienced some improvement in mental health, physical or social functioning (assessed by the SF-36) or an improvement in one of the specific back pain categories (at rest, during the day, at night, or on movement). Patients who had no response in any aspect tended to withdraw from the study after a short period (median time to withdrawal was 15 days for non-responders vs 99 days for others, mean 34 and 139 days, respectively, p < 0.001). Only 10 (7%) of these non-responders completed the study. The commonest reason for withdrawal was an adverse event that occurred in 111/136 (82%) of the dropouts.

The small group of non-responders who remained in the study for more than 4 weeks (n = 37) tended to be on higher opioid doses than other patients. The mean dose (mg morphine or equivalents for fentanyl) at 5 weeks was 116 (95% CI 92, 139) mg for non-responders compared with 94 (89, 98) mg for other participants. At 12 weeks, the mean doses were 156 (95% CI 104, 207) mg and 114 (95% CI 107, 120) mg, respectively.

Some aspects of pain improved more than others with strong-opioid treatment, e.g. the responders reporting severe pain on movement fell from 66% at baseline to 21% at endpoint.

### Effects of age

Older patients (defined as those at least 75 years old, n = 51) tended to use lower opioid doses than younger patients (<75 years, n = 621). The median daily doses at study endpoint were 58 mg for SRM and 43 μg/h (102 mg) for TDF for older patients compared with 116 mg SRM and 54 μg/h (130 mg) TDF for younger patients. The average morphine dose in older patients remained stable throughout the study; the mean morphine dose was 57 mg at baseline and 59 mg at endpoint, however the mean dose in patients who completed the study was 66 mg at month 13. The average fentanyl dose was 25 μg/h at baseline, 44 μg/h at endpoint (for all patients, whenever they left the study), and 50 μg/h at month 13 (i.e. for patients who completed the study).

The proportion of responders tended to be higher in the younger than the older age groups (56% vs 45%, respectively, for 30% response and 40% vs 31%, respectively, for 50% response). However, no statistical significance could be detected, probably due to the small sample size. The proportion of patients who withdrew because of adverse events was higher in the elderly sub-group than in the younger patients: rates of withdrawal were 56% (n = 15) for SRM and 42% (n = 10) for TDF in the older group compared with 29% (n = 89) and 37% (n = 115), respectively in the younger patients.

### Adverse events

**Figure 3:**
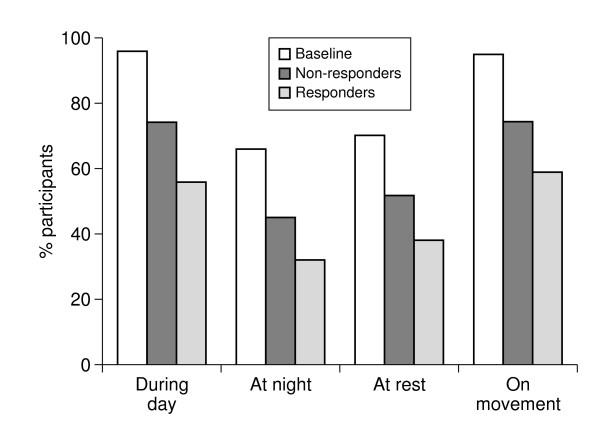
Proportion of patients with moderate/severe pain at baseline and endpoint by response category.

Most adverse events (AEs) were reported in the first few weeks of the study, except for sweating in the fentanyl group, which increased over time (Figure 3). To examine whether patients who experienced AEs were more likely to withdraw from the study before an effective dose had been reached, we compared doses in patients with and without the most common AEs.

The median daily opioid dose was lower for patients <75 years old who reported nausea than for those who did not (76 mg morphine (n = 159) or 38 μg fentanyl (n = 170) for patients with nausea vs 85 mg morphine (n = 151) or 48 μg fentanyl (n = 141) for those without). This pattern was not seen for those who reported constipation (median morphine dose of SRM patients <75 years was 84 mg for those with (n = 204) vs 72 mg for those without (n = 106) constipation, (doses for fentanyl 48 μg (n = 163) vs 38 μg (n = 148), respectively).

Evidence that vomiting may limit treatment comes from the observation that patients who experienced at least a 30% pain relief were less likely to report this AE than non-responders (23% of responders and 33% of non-responders reported vomiting). However responders were more likely to report constipation than non-responders (67% vs 47%), perhaps reflecting the greater length of time they remained in the study since constipation tends to occur after several weeks of opioid treatment.

### Dosage and study completion

Dose increases between 4 weeks and endpoint tended to be larger in patients who completed the study than those who dropped out. For example, the proportion of patients whose opioid dose more than doubled during this time was 24% for completers and 7% for non-completers.

### Best and worst responders

Demographic and baseline characteristics of the best and worst responders (top and bottom 15%) were compared. The mean (±standard error of the mean (SEM)) duration of chronic pain was no different in the poor responders than in the best responders (106 ± 10 vs 94 ± 8 months).

No clear patterns emerged that might predict response, except that there were significantly more women in the SRM worst response group (73% of the poorest responders to morphine were female compared with 49% of the poorest responders to fentanyl, p = 0.017).

### Reasons for leaving study

Patients who left the study in the first month (i.e. early drop-outs, n = 135 comprising 20% of the total population) were compared with those who withdrew from the study at a later stage (n = 199). Drop-outs within the first month were more likely to be due to adverse events (82% vs 59%) and less likely to be due to insufficient response (3% vs 15%) than with later withdrawals. The proportion of patients withdrawing consent was 9% among early dropouts and 6% among later withdrawals.

Adverse events during the first month were compared between early dropouts and all patients who remained in the study for longer (n = 540). Nausea and vomiting were more common in early dropouts (62% vs 43% for nausea, and 38% vs 20% for vomiting, respectively). Constipation and increased sweating were less frequent among early dropouts (36% vs 49% for constipation, and 8% vs 14% for sweating). Pruritus was similar in early dropouts and other patients (12% and 17%, respectively).

### Effects of dose

The effect of opioid dose was assessed by considering the sub-group of patients who received higher doses (>100 μg/h TDF or >390 mg/day SRM, n = 43). The incidence of adverse events was similar in both dosage groups, except for sweating, which occurred more often in the higher dose group (35% higher dose vs 20% lower dose, p = 0.0207). The proportion of patients achieving at least 30% pain relief was higher in the higher dose group (70% vs 54%, p = 0.043). A similar pattern was observed for the proportions achieving at least 50% pain relief, but the difference between dose groups (44% vs 39%) did not reach statistical significance when both treatments were considered together. There was a marked difference in the proportion of patients achieving at least 50% pain relief in the higher dose sub-group between those receiving TDF (52% responders) and those receiving SRM (29%). This difference was not observed in the lower dose group (39% responders in each treatment group).

### Multivariate analysis

We performed a step-wise logistic regression using 13 key variables (Table 2). No strong correlation between any covariates was found. The most influential factors for predicting a response of at least a 30% reduction of pain from baseline were employment status (χ^2 ^= 11.06, p = 0.0259) and use of high doses of opioids (χ^2 ^= 3.04, p = 0.0811). The category 'non-professional' employment status, which included housewives but not unemployed, retired people or students, was associated with a lower likelihood of achieving a 30% response than the 'disabled' category (odds ratio compared with disabled category 0.412 (95% CI 0.24 to 0.70) p = 0.0010). The use of higher opioid doses during the trial gave an increased likelihood of achieving a 30% response. This effect was statistically significant (p = 0.049) in the first model, which included all covariates, but fell below statistical significance in the final model (p = 0.081, odds ratio 1.828 (95% CI 0.93 to 3.60)).

**Table 2:** Logistic regression on the probability of 30% responseNegative values indicate reduced probability of 30% response.

**Variable (baseline value)**	**Effect estimate**	**p Value of Type III effect**	**Category (p value)**	**Odds ratio**	**95% CI**
Age	-0.0007	0.9350		0.999	0.98–1.02
Mental health	0.0045	0.2935		1.004	1.00–1.01
Pain at rest	-0.3612	0.1779	Moderate^1 ^(0.0633)	0.697	0.48–1.02
Compared with no/slight pain	-0.2503		Severe^1 ^(0.2783)	0.779	0.50–1.22
Pain on movement	-0.1683	0.6717	Moderate^2 ^(0.6788)	0.845	0.38–1.87
Compared with no/slight pain	-0.2926		Severe^2 ^(0.4652)	0.746	0.34–1.64
Physical functioning (SF-36)	-0.0055	0.2270		0.995	0.99–1.00
Social functioning (SF-36)	-0.0036	0.3129		0.996	0.99–1.00
Duration of pain	-0.0006	0.4152		0.999	0.99–1.00
Education	-0.2680	0.5083	Apprenticeship^3 ^(0.3462)	0.765	0.44–1.34
Compared with primary education	0.0649		Secondary education^3 ^(0.7356)	1.07	0.73–1.56
	0.2464		Higher education^3 ^(0.4168)	1.28	0.71–2.32
Employment	-0.1039	0.0582	Employed^4 ^(0.6751)	0.901	0.55–1.47
Categories compared with disabled	-0.8852		Non-professional^4 ^(0.0029)	0.413	0.23–0.74
	-0.2908		Retired^4 ^(0.2201)	0.748	0.47–1.19
	-0.2739		Student^4 ^(0.4163)	0.760	0.39–1.47
High dose	0.7185	0.0499		2.051	1.00–4.21
Neuropathic pain	-0.1857	0.2735		0.831	0.60–1.16
Treatment group	0.0530	0.7434		1.054	0.77–1.45
Sex	0.0439	0.8033		1.045	0.74–1.48

Variables predicting at least a 50% reduction in pain were similar, and employment status (and the non-professional category) was one of the most influential factors, however its contribution was not statistically significant (p = 0.127). The strongest predictor for 50% response was the presence of neuropathic pain, which was weakly predictive of poorer response (p = 0.0276). The odds ratio for the presence of neuropathic pain on the likelihood of 50% response was 0.695 (95% CI 0.50 to 0.96).

## Discussion

By 1 month, 20% of patients had stopped treatment, and four out of five stopped treatment because of adverse events. However, 54% of patients had at least 30% pain relief during one time point of the trial and 69% of these patients were still on opioid treatment after 12 months, the dosages used by responders did not differ from those used by non-responders.

This study provided an opportunity to investigate predictive factors for response and gives important information regarding the efficacy and natural course of adverse effects over 13 months. However, this study was not originally designed to test predictors and therefore many potentially important factors were not examined (e.g. motivation and expectations of the patient and the physician, and the impact of other medications). As the study included only carefully selected patients with a long history of chronic low back pain, the findings need to be verified in a broader population.

No clear pattern of baseline pain (type or severity) or patient characteristics (e.g. age, gender) emerged that could be used to predict responders before the start of opioid treatment. However, this analysis did suggest that a 1-month trial period is sufficient to determine response and tolerability in most cases. The significant relationship between response at 1 month and response after 6 and 12 months suggests that if patients have not responded to a suitable dose of strong opioid after 1 month, then treatment should be stopped and alternative pain relief introduced. However, if patients experience at least a 30% reduction in pain at 1 month, then opioid treatment should be continued, as long-term benefit is more likely. Previous studies have also found an association between response to intravenous opioids and long-term treatment [13]. As in the present study, Dellemijn et al. found that not all patients responding to a single dose of an intravenous opioid responded as well to long-term treatment. The negative predictive power was stronger, and those who did not respond to the intravenous opioid were less likely to respond to long-term transdermal treatment.

Our findings can be expressed in terms of the sensitivity and specificity of response at 1 month to predict response at 6 months. Specificity is the probability of a response at 1 month and 6 months (i.e. the true positive rate) and is calculated to be 0.505. Sensitivity is the probability of no response at 1 month for those with no response at 6 months (i.e. the true negative rate) and is calculated to be 0.810. Thus lack of response at 1 month has stronger negative predictive power than the positive predictive power of the presence of response at 1 month.

We noted that there were significantly more women in the SRM worst response group than in the equivalent group for TDF (73% vs 49%). Whether this is an indicator of sex specific differences in response to opioid treatment needs further investigation.

This large dataset allowed us to examine correlations between the main outcome of the original study (pain relief expressed by a decrease in VAS pain scores) and other measurements of pain. We found a statistically significant correlation between the pain relief score and the change in back pain category (mild, moderate or severe) from baseline to study endpoint.

Gastrointestinal side effects are relatively common in patients receiving strong opioids for the first time. Nausea and vomiting appear to limit treatment in some cases, and may prevent titration to effective doses. Constipation tends to emerge after a few weeks of treatment, and occurred more frequently in patients receiving morphine than in those receiving transdermal fentanyl. The median daily opioid dose was lower in patients who reported nausea than in those who did not but this pattern was not seen for constipation. This suggests that the occurrence of nausea during the early weeks of treatment may limit dose titration, whereas constipation generally emerges later. The higher median dose of TDF in patients who reported constipation compared with those that did not might also reflect a dose-response effect, with constipation occurring only with higher doses of TDF.

The rate of adverse effect reporting is affected by the reporting method. In our study, only adverse events volunteered by patients (or observed by clinicians) were reported, whereas, in other studies, patients have been questioned specifically about the occurrence of expected side effects. This may explain why the rates of adverse events in our study are lower than those in some previous studies of strong opioids [14]. However the adverse events in this trial were analyzed with care.

Patients aged over 75 years in the SRM group showed stable doses throughout the study, in contrast to older patients receiving TDF and to the younger patients in both groups. Older patients in the SRM group reported less pain relief than younger patients; this might also suggest that effective morphine doses were not reached. One possible explanation for this is that older patients are more susceptible to dose-related morphine side effects and this limited dose escalation. This assumption is supported by the higher dropout rate in the older patient group (56% in the TDF and 42% in the SRM group). Other studies have also noted that morphine side effects may be more pronounced in elderly patients [15].

Only a small proportion of patients needed high opioid doses, but these patients tended to achieve additional pain relief.

Patients who experienced effective pain relief (e.g. a decrease of at least 30% from baseline) tended to remain in the trial longer than those who did not get adequate analgesia. Some patients may have remained in the trial because of other benefits apart from the primary outcome, such as sleeping better. A few patients, who according to trial assessments had experienced no benefits, still completed the 13-month trial.

## Conclusion

Strong opioid treatment can be beneficial for some patients with severe low back pain. However, there appears to be no simple method of predicting who will benefit from a strong opioid before treatment is initiated. This analysis suggests that response to a trial period of 1 month gives a good indication of who will obtain sustained benefit. Neuropathic pain does not appear to rule out response to strong opioids, but it may be associated with a poorer response. Gastrointestinal side effects are common during the first weeks of treatment, and these may limit dose titration in some patients. The best way to determine who will tolerate a strong opioid appears to be a trial period of 1 month.

## Competing interests

The original study (FEN-INT-26) and the secondary analysis were funded by Janssen Pharmaceutica, the manufacturer of transdermal fentanyl. EK, KHS and RS were investigators in the original study and received funding from Janssen Pharmaceutica for this. They have also worked with Janssen on other clinical trials and received research funding and honoraria at various times. UR is an employee of Janssen Cilag.

## Authors' contributions

EK and UR had the original idea for this secondary analysis. JD performed the statistical analyses. EK, KS, RS, DJ and UR all reviewed and revised several drafts of the manuscript and provided critical comments. All authors read and approved the final manuscript.

## Pre-publication history

The pre-publication history for this paper can be accessed here:


